# Design-features of bubble-prone experimental asset markets with a constant FV

**DOI:** 10.1007/s40881-019-00061-5

**Published:** 2019-03-26

**Authors:** Christoph Huber, Parampreet C. Bindra, Daniel Kleinlercher

**Affiliations:** 1 Department of Banking and Finance, University of Innsbruck, Universitätsstrasse 15, 6020 Innsbruck, Austria; 2 Department of Economics, University of Innsbruck, Universitätsstrasse 15, 6020 Innsbruck, Austria

**Keywords:** Experimental finance, Asset markets, Price efficiency, Bubbles, Experimental design, C92, D84, G02, G12, G14

## Abstract

Experimental asset markets with a constant fundamental value ($\mathrm {\textsc {fv}}$) have grown in importance in recent years. A methodological examination of the robustness of experimental results in such a setting which has been shown to produce bubbles, however, is lacking. In a laboratory experiment with 280 subjects, we investigate whether specific design features are sufficient to influence experimental results. In detail, we (1) vary the visual representation of the price chart, and (2) provide subjects with full information about the FV process. We find overvaluation and bubble formation to be reduced when trading prices are displayed at the upper end of the price chart. Surprisingly, we do not find any effects when subjects have full information about the FV process.

**Electronic supplementary material:**

The online version of this article (10.1007/s40881-019-00061-5) contains supplementary material, which is available to authorized users.

## Introduction

Experimental design-features are important issues concerning methods for laboratory asset markets and are crucial for the interpretation of experimental results. From a methodological standpoint, the seminal design of Smith et al. (1988, $\mathrm {\textsc {ssw}}$ henceforth) has been thoroughly examined over the last decades with evidence that seemingly small variations in the experimental design can matter a lot.^1^ It has been argued that price bubbles in decreasing fundamental value ($\mathrm {\textsc {fv}}$) designs are a result of the mismatch between the asset’s $\mathrm {\textsc {fv}}$ trajectory and subjects’ expectations of a non-decreasing price development (e.g. Smith 2010; Oechssler 2010). To circumvent this mismatch, experimental asset market designs with constant $\mathrm {\textsc {fv}}$s have been implemented more frequently in the last years (see e.g. Kirchler et al. 2015; Razen et al. 2017; Holt et al. 2017; Weitzel et al. 2018). With the increasing popularity of constant $\mathrm {\textsc {fv}}$ designs, it is also increasingly important to examine the characteristics of such an experimental design. However, a methodological analysis for experimental settings with constant $\mathrm {\textsc {fv}}$ regimes is missing at the moment.

The goal of this paper is to investigate whether certain design-features can influence results in experimental asset markets with a constant $\mathrm {\textsc {fv}}$ regime. We specifically examine the experimental asset market design put forward by Holt et al. (2017), which has increasingly been applied in the last years and has been shown to produce typical bubble-crash patterns (Giusti et al. 2016; Weitzel et al. 2018). We therefore employ a continuous double auction market for long-lived assets with dividend and interest payments, exogenous cash inflows, and a constant $\mathrm {\textsc {fv}}$ trajectory.^2^

In this setting we examine whether two seemingly irrelevant, experimental design-features affect experimental results: First, we manipulate the display of trading prices in the price chart during and after trading periods. While, in general, graphical distortions in information processing have been widely discussed (e.g. Tufte 1983; Beattie and Jones 1992), there is also evidence that in market settings already a different presentation of trading prices and the $\mathrm {\textsc {fv}}$ prior to the experiment can influence experimental results. Cason and Samek (2015) for example find that the visual representation of trading prices—either displayed in a column of text or in a graphical display—leads to significantly different price levels. Huber and Kirchler (2012) demonstrate that bubble formation is significantly reduced when the $\mathrm {\textsc {fv}}$ process is displayed in a graph instead of a table prior to the experiment. Baghestanian and Walker (2015) show that setting a visual anchor at the $\mathrm {\textsc {fv}}$ in the price chart at the beginning of the experiment is sufficient to eliminate or to significantly reduce bubbles. These studies, however, are only concerned with decreasing $\mathrm {\textsc {fv}}$ regimes. Regarding price charts in general, Lawrence and O’Connor (1993) argue that with smaller scales, prediction intervals get wider and the scale might influence subjects’ perception of variability. Huber and Huber (2019) confirm this intuition and report that the vertical axis scale strongly affects people’s risk perception such that price developments are perceived as riskier when the depicted line extends to the upper or lower borders of a chart. In a similar vein, we alter the vertical axes of price charts during trading across treatments. From a baseline treatment with a standard axis around the middle of the scale we derive two treatments by varying the scale to induce either a high or a low anchor. With these treatment variations, we can detect whether results are driven by seemingly irrelevant display choices and, thus, hint at confusion among subjects in this experimental setting.

Second, we provide subjects with full information about the $\mathrm {\textsc {fv}}$ trajectory which includes detailed explanations in the instructions and a training protocol prior to the experiment. We follow Caginalp et al. (2001), Dufwenberg et al. (2005), Ackert et al. (2006), Noussair et al. (2012), and Giusti et al. (2016), among others, in displaying the $\mathrm {\textsc {fv}}$ development over time in a table. Here, we aim to rule out confusion among subjects as we provide information about the $\mathrm {\textsc {fv}}$ upfront and for any given point in time during the experiment. This treatment builds on research that shows that unambiguity and common knowledge about the dividend structure and thereby about the $\mathrm {\textsc {fv}}$ process is able to reduce bubbles (Lei and Vesely 2009; Kirchler et al. 2012; Cheung et al. 2014) in $\mathrm {\textsc {ssw}}$-like experimental asset markets. Finally, we want to stress that the treatment manipulations in this study address important experimental design choices every researcher has to make when designing a laboratory asset market experiment. On one side, we test how sensitive subjects react to different visual stimuli and, on the other, how sensitive subjects react to information about the $\mathrm {\textsc {fv}}$ process.

We observe significant overpricing and typical bubble-crash patterns in all treatments, though with differences of overvaluation across treatments. We find that overvaluation and typical bubble-crash patterns are reduced when prices are displayed in the upper third of the price chart and thereby induce a visual ceiling. While such a result hints at confusion about the $\mathrm {\textsc {fv}}$ among subjects, surprisingly, we do not find subjects’ common knowledge about the $\mathrm {\textsc {fv}}$ process to reduce overvaluation.

## The experiment

### Market design and fv process

We implement an asset market environment which is related to the designs of Smith et al. (2014), Holt et al. (2017), and Weitzel et al. (2018). In a continuous double auction market setting, eight subjects trade assets of a fictitious company for experimental currency units (Taler) in a sequence of 20 periods of 120s each. At the beginning of the market, every subject is endowed with 20 assets and 560 Taler. One unit of the asset pays a dividend of 1.20 or 1.60 Taler with equal probability at the end of each period. The dividend is independently drawn for every period and is the same for all assets in a given period. Cash held after market transactions (but before dividend payments) pays 5% of interest. Dividends and interest on Taler holdings are paid at the end of each period. Taler and stock holdings are then carried over from one period to the next. At the end of the experiment each unit of the asset pays a redemption value of 28 Taler. At the start, the total cash amount in the market ($8 \times 560\,{\text {Taler}} = 4480\,{\text {Taler}}$) is equal to the value of all outstanding assets in the market ($8 \times 20 = 160\,{\text {assets}}, 160 \times 28\,{\text {Taler}} = 4480\,{\text {Taler}}$); hence, the initial cash-to-asset ratio ($\mathrm {\textsc {ca}}$ ratio) is 1. Due to an exogenous cash inflow of 100 Taler, dividends of 1.20 or 1.60 Taler, and interest payments of 5% on Taler holdings each period, the $\mathrm {\textsc {ca}}$ ratio increases from initially 1 to roughly 4.1 in Period 10, and 10.2 at the end of trading after Period 20. No new assets are issued at any time.

To determine the asset’s $\mathrm {\textsc {fv}}$, subjects know the interest rate on Taler holdings *r*, possible dividend realizations ${\tilde{d}}$, their probability of occurrence, the total number of trading periods *T*, and the redemption value of the asset *K*. The $\mathrm {\textsc {fv}}$ at the beginning of period *t* is calculated as the net present value of all remaining dividend payments and the redemption value at the end of *T*, i.e.

1 $$\begin{aligned} FV_t= & {} \, E({\tilde{d}}) \biggl [ \sum _{\tau =1}^{T-t+1} (1+r)^{-\tau }\biggr ] + K (1+r)^{-(T-t+1)} \end{aligned}$$

2 $$\begin{aligned}= & {} \, E({\tilde{d}})/r + (K-E({\tilde{d}})/r)(1+r)^{-(T-t+1)} \quad {\text {if}}\quad r \ne 0. \end{aligned}$$

The time trend of the $\mathrm {\textsc {fv}}$ is then given by

3 $$\begin{aligned} \frac{d(FV_t)}{dt}= & {} \, (K-E({\tilde{d}})/r)[\ln (1+k)](1+r)^{-(T-t+1)} \quad {\text {if}}\quad r \ne 0. \end{aligned}$$

As in Holt et al. (2017), we set $K=E({\tilde{d}})/r$ with $K,\, E({\tilde{d}}),\, r > 0$ to induce a constant $\mathrm {\textsc {fv}}$ process. To see this, consider Eqs. (2) and (3) from above, which then reduce to

$$\begin{aligned} FV_t&= E({\tilde{d}})/r \quad {\text { (2a)}} \quad {\text {and}} \quad \frac{d(FV_t)}{dt} =0. \quad {\text {(3a)}} \end{aligned}$$

The intuition behind this derivation is that the redemption value *K* represents the discounted expected value of all dividends that would have been received after the final period if the experiment had lasted indefinitely, i.e., the present value of a perpetuity paying the expected dividend $E({\tilde{d}})$ in each period (see Bostian et al. 2005).^3^

### Price beliefs

We elicit subjects’ beliefs about market prices. With this approach we investigate whether potential bubbles also operate on the level of beliefs. In detail, subjects are asked to forecast the average market prices for the following three periods, i.e., in every period *t* we elicit price beliefs for periods $t+k$ with $k=0,\,1,\,2$. In periods 19 and 20 we only elicit beliefs for the remaining periods.^4^ For every forecast within $\pm 5\%$ of the ex-post observed price subjects earn 50 Taler.

### Treatments

In Treatment $\mathrm {\textsc {base}}$, we do not manipulate the price chart during trading periods and after trading in the history screen. Thus, the price chart presents the maximum trading price within a period in the middle third of the vertical axis. Furthermore, we do not provide the subjects with full information about the formation of the $\mathrm {\textsc {fv}}$.

In Treatment $\mathrm {\textsc {ceiling}}$, we alter the visual representation of the price development both within a period on the trading screens and between periods on the history screens. In particular, the vertical axes of the price charts are adjusted to show the highest price in a period in the upper third of the scale. Here, having prices at the upper end of the scale might be viewed as a visual ‘*ceiling*’ and suggests that the price is already at a considerably high level.

In Treatment $\mathrm {\textsc {floor}}$, we vary the scale in the opposite direction, i.e., depicting the highest price in a period in the lower third of the scale. Here, the price is always displayed close to the ‘*floor*’ of a price chart, which hints at the price being at a comparatively low level.

Treatment $\mathrm {\textsc {info}}$ resembles Treatment $\mathrm {\textsc {base}}$ with the only exception that we provide subjects with full information about the $\mathrm {\textsc {fv}}$. In detail, we include a table presenting the composition of the $\mathrm {\textsc {fv}}$ in each period and provide an example for calculating the $\mathrm {\textsc {fv}}$ in a given period in the experimental instructions. Furthermore, subjects have to complete a training task, which consists of correctly entering the $\mathrm {\textsc {fv}}$ of the asset for each period. This procedure ensures that all subject have full information about the $\mathrm {\textsc {fv}}$ and about other participants’ knowledge about the $\mathrm {\textsc {fv}}$ at any time during the experiment.

**Fig. 1:**
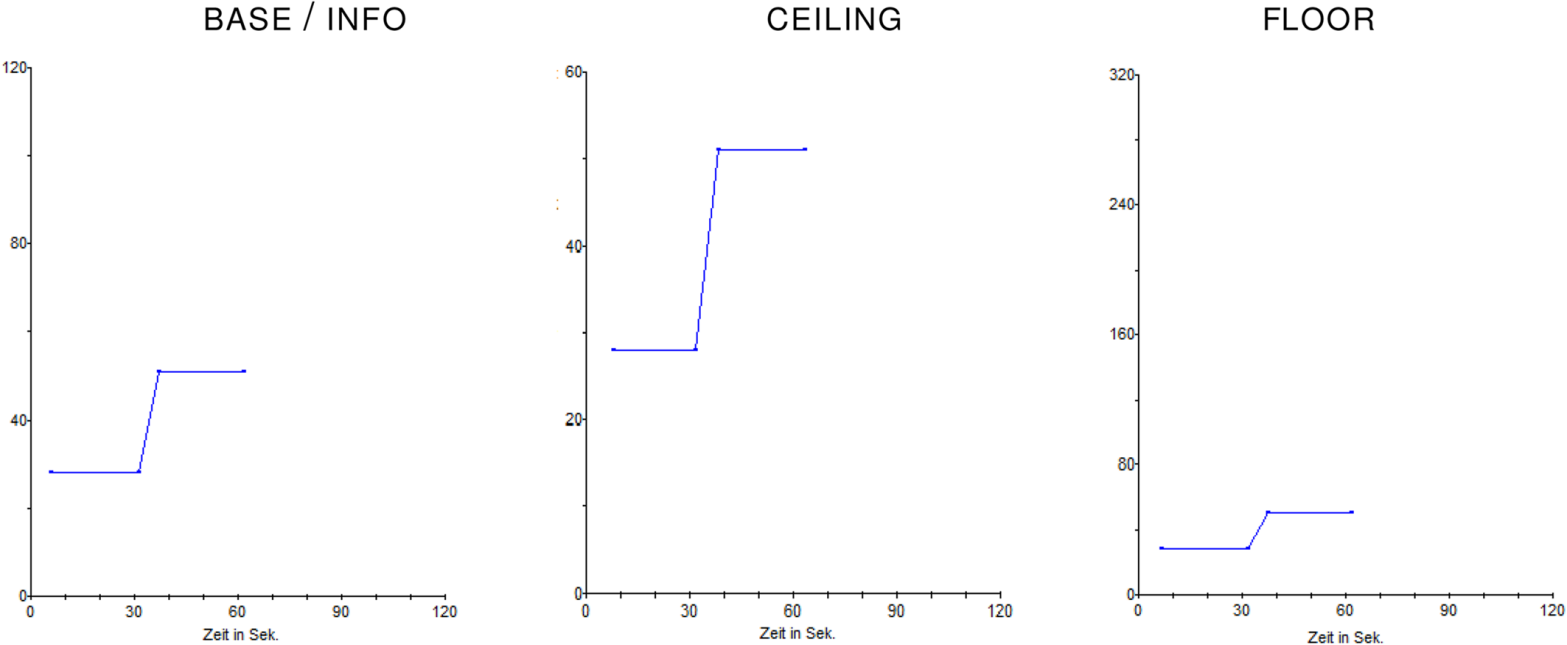
Exemplary price charts displayed on the trading screens in treatments $\mathrm {\textsc {base}}$ and $\mathrm {\textsc {info}}$ (left), $\mathrm {\textsc {ceiling}}$ (middle) and $\mathrm {\textsc {floor}}$ (right) for a sample maximum price of 51

Figure 1 depicts exemplary price charts for a maximum price of 51 Taler for treatments $\mathrm {\textsc {base}}$ and $\mathrm {\textsc {info}}$ (left), $\mathrm {\textsc {ceiling}}$ (middle), and $\mathrm {\textsc {floor}}$ (right).^5^ In each treatment the axes’ scales were carefully designed to match specific criteria. In treatments $\mathrm {\textsc {base}}$ and $\mathrm {\textsc {info}}$ there are never less than 50 percent and on average 56 percent of the axis above the maximum price. In $\mathrm {\textsc {ceiling}}$ there are never more than 24 percent and on average six percent of the axis above the maximum price (assuming prices between 10 and 400 Taler); in $\mathrm {\textsc {floor}}$ there is always at least 75 percent and on average 79 percent of the axis above the maximum price.

### Experimental implementation

We ran nine markets each for treatments $\mathrm {\textsc {base}}$, $\mathrm {\textsc {info}}$, and $\mathrm {\textsc {ceiling}}$; and eight markets for Treatment $\mathrm {\textsc {floor}}$. All 35 markets were conducted between April 2016 and February 2017 at Innsbruck EconLab at the University of Innsbruck with a total of 280 students (mostly bachelor and master students in business administration and economics). The markets were programmed and conducted with z-Tree by Fischbacher (2007) and GIMS by Palan (2015). Subjects were recruited using hroot by Bock et al. (2014).

In total, each experimental session lasted approximately 105 minutes. This included 10 minutes to study the written instructions, a detailed explanation of the trading screen, two trial periods, and the market experiment. Additionally, subjects participated in a risk experiment, i.e., a variation of the bomb risk elicitation task (BRET; Crosetto and Filippin 2013), prior to the market experiment, though the results from the risk experiment were revealed after the market experiment. At the end of the experimental session, subjects had to complete a questionnaire assessing their understanding of the $\mathrm {\textsc {fv}}$ process and their score in a Cognitive Reflection Test (CRT; Frederick 2005), collecting demographic data, as well as eliciting risk attitudes with a survey question from the German Socio-Economic Panel (SOEP; Dohmen et al. 2011) and a question concerning investment decisions.

Subjects’ payout comprises earnings from the risk experiment and of earnings from the market experiment including the belief elicitation task. For the market experiment, the redemption value of the asset was multiplied by a subject’s units of the asset held at the end of the experiment and added to the end holdings in Taler. Finally, the amount of Taler was exchanged for euros at a conversion rate of 400:1 in all treatments. Average payouts were 20.70 euros with a standard deviation of 5.12 euros.

## Results: overvaluation and bubble formation

**Fig. 2:**
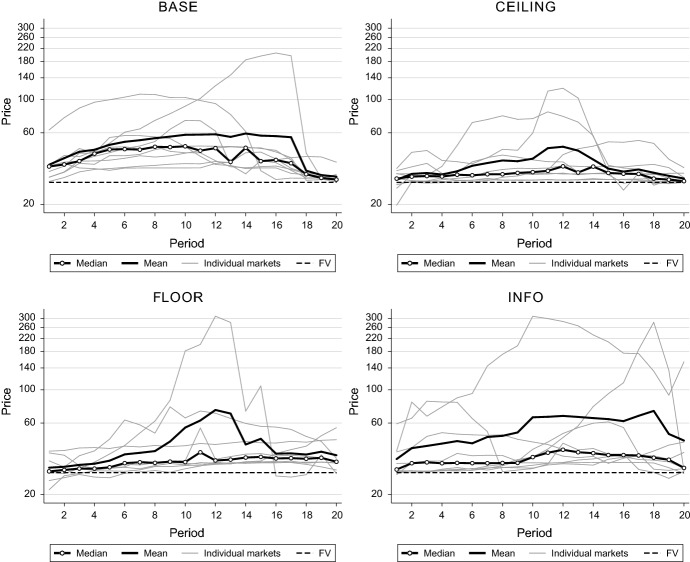
Median treatment prices (bold and colored lines with circles), mean treatment prices (bold and colored lines without circles), volume-weighted mean prices for individual markets (grey lines), and the fundamental value ($\mathrm {\textsc {fv}}$, dashed line) as a function of period for treatments $\mathrm {\textsc {base}}$ (top left), $\mathrm {\textsc {ceiling}}$ (top right), $\mathrm {\textsc {floor}}$ (bottom left), and $\mathrm {\textsc {info}}$ (bottom right)

Figure 2 outlines average (volume-weighted) price developments across periods of individual markets as well as treatment medians and means for each of the four treatments. Overall, all treatments exhibit strong price increases with falling market prices toward the end of the experiment, i.e., we observe typical bubble and crash patterns across all treatments. We use relative deviation ($\mathrm {\textsc {rd}}$; Stöckl et al. 2010), which is calculated by averaging differences between the volume-weighted mean price and the $\mathrm {\textsc {fv}}$ across all periods and normalizing it with the absolute value of the average $\mathrm {\textsc {fv}}$ of the market, as a measure for overvaluation.^6^

**Result 1** In all treatments, we observe significant levels of overvaluation and typical bubble and crash patterns. Even with full information about the $\mathrm {\textsc {fv}}$, price inefficiencies remain.

*Support* Each treatment’s $\mathrm {\textsc {rd}}$ is significantly different from zero (Wilcoxon signed-rank tests, $p<0.01$), suggesting a positive relative price deviation from the $\mathrm {\textsc {fv}}$. Thus, we observe the typical overvaluation of prices in this bubble-prone experimental asset market design and neither of our treatment manipulations is sufficient to completely eliminate price inefficiencies.

As a next step, we investigate whether we can detect differences between treatments in important market variables.

Therefore, we run pairwise comparisons between treatments for relative deviation ($\mathrm {\textsc {rd}}$), share turnover ($\mathrm {\textsc {st}}$), volatility (the standard deviation of log-returns, $\mathrm {\textsc {vola}}$), and the bid-ask spread ($\mathrm {\textsc {spread}}$).

**Result 2** By manipulating the vertical axis of the price charts in Treatment $\mathrm {\textsc {ceiling}}$, $\mathrm {\textsc {rd}}$ is considerably reduced compared to Treatment $\mathrm {\textsc {base}}$ but not compared to Treatment $\mathrm {\textsc {floor}}$. In contrast, ensuring that subjects have full information about the $\mathrm {\textsc {fv}}$ (Treatment $\mathrm {\textsc {info}}$) does not reduce $\mathrm {\textsc {rd}}$. Other market variables exhibit no differences.

*Support* Treatment $\mathrm {\textsc {base}}$ exhibits a median relative deviation ($\mathrm {\textsc {rd}}$) as a percentage of the $\mathrm {\textsc {fv}}$ of 47.3%. The manipulation of the vertical axis in Treatment $\mathrm {\textsc {ceiling}}$ lowers $\mathrm {\textsc {rd}}$ to 16.9% and Treatment $\mathrm {\textsc {floor}}$ leads to a median $\mathrm {\textsc {rd}}$ of 21.3%. Thus, in Treatment $\mathrm {\textsc {ceiling}}$, $\mathrm {\textsc {rd}}$ is reduced by 30.4 percentage points compared to Treatment $\mathrm {\textsc {base}}$ (Mann–Whitney *U*-test, $p = 0.038$).^7^ Thus, it seems that subjects are indeed influenced by the manipulation of the vertical axis and do not trade assets at higher levels which we observe in the other treatments. In addition, we find no indication that $\mathrm {\textsc {rd}}$ is increasing in the treatment—i.e., in the vertical axis scale—in the order $\mathrm {\textsc {ceiling}}$, $\mathrm {\textsc {base}}$, $\mathrm {\textsc {floor}}$ (Jonckheere trend test, $p = 0.233$). Turning to Treatment $\mathrm {\textsc {info}}$, where subjects have full information about the $\mathrm {\textsc {fv}}$, we observe $\mathrm {\textsc {rd}}$ to be similarly strongly pronounced as in $\mathrm {\textsc {base}}$ (47.1 vs. 35.7%, $p = 0.402$); hence, we observe no improvement regarding market efficiency when providing subjects with full information about the $\mathrm {\textsc {fv}}$ (Table 1).

**Table 1:** Treatment medians of market variables and pairwise comparisons

Treatment	$\mathrm {\textsc {rd}}$	$\mathrm {\textsc {st}}$	$\mathrm {\textsc {vola}}$	$\mathrm {\textsc {spread}}$	
$\mathrm {\textsc {base}}$	47.32	30.62	16.77	19.64	
$\mathrm {\textsc {ceiling}}$	16.91	25.63	16.55	25.00	
$\mathrm {\textsc {floor}}$	21.26	23.75	12.93	11.43	
$\mathrm {\textsc {info}}$	35.65	23.13	18.23	23.21	

Pairwise MW *U*-tests	$\mathrm {\textsc {rd}}$	$\mathrm {\textsc {st}}$	$\mathrm {\textsc {vola}}$	$\mathrm {\textsc {spread}}$	*N*
$\mathrm {\textsc {base}}$ versus $\mathrm {\textsc {ceiling}}$	2.075**	0.707	0.397	− 0.089	18
$\mathrm {\textsc {base}}$ versus $\mathrm {\textsc {floor}}$	1.443	0.963	0.385	1.157	17
$\mathrm {\textsc {ceiling}}$ versus $\mathrm {\textsc {floor}}$	− 0.674	0.289	− 0.192	1.110	17
$\mathrm {\textsc {base}}$ versus $\mathrm {\textsc {info}}$	0.839	0.840	0.397	0.000	18

*Top panel:* Treatment medians of relative deviation ($\mathrm {\textsc {rd}}$), share turnover ($\mathrm {\textsc {st}}$), the standard deviation of log-returns ($\mathrm {\textsc {vola}}$, and the bid-ask-spread at the end of a period ($\mathrm {\textsc {spread}}$). *Bottom panel:* Significance tests for treatment differences. The numbers indicate *Z*-values of pairwise Mann–Whitney *U*-tests

*, **, and *** represent *p* values smaller than 0.10, 0.05, and 0.01, respectively, for double-sided tests

After having investigated overall price levels and other market variables across treatments, we now examine whether there are differences in bubble formation. Therefore, we follow Razen et al. (2017) and use $\mathrm {\textsc {rdmax}}$ as a measure for overvaluation at the peak price, $\mathrm {\textsc {amplitude}}$ as a measure for price run-ups before the peak price, and $\mathrm {\textsc {crash}}$ as a measure of the magnitude of price downturns after the peak.^8^ The top panel of Table 2 shows median values of the respective variables across treatments. To test for differences between treatments, we use pairwise Mann–Whitney *U*-tests which are outlined in the middle panel of Table 2.

**Result 3** Treatment $\mathrm {\textsc {ceiling}}$ exhibits the least-pronounced values across all bubble measures; the other treatments show up to more than two times higher numbers. Full information about the $\mathrm {\textsc {fv}}$ (Treatment $\mathrm {\textsc {info}}$) is not sufficient to considerably reduce any of the measures.

*Support* From the top panel of Table 2 representing treatment medians, one can clearly see that $\mathrm {\textsc {rdmax}}$ (31.9%), $\mathrm {\textsc {amplitude}}$ (39.5%), and $\mathrm {\textsc {crash}}$ (-24.3%) are lowest in Treatment $\mathrm {\textsc {ceiling}}$. The remaining three treatments show considerably inflated values for all bubble measures, reflecting the differences to $\mathrm {\textsc {ceiling}}$ we observe visually in Fig. 2 above. While median values suggest that the respective bubble identification measures increase in the vertical axis scale in the treatment order $\mathrm {\textsc {ceiling}}$ (smallest values), $\mathrm {\textsc {base}}$ (intermediate), $\mathrm {\textsc {floor}}$ (largest values), Jonckheere tests show no trend (*p* values between 0.13 and 0.26). Regarding Treatment $\mathrm {\textsc {info}}$, we again find no improvement—i.e., lower values in bubble identification measures—compared to Treatment $\mathrm {\textsc {base}}$.

**Table 2:** Treatment medians of bubble identification measures and pairwise comparisons

Treatment	$\mathrm {\textsc {rdmax}}$	$\mathrm {\textsc {amplitude}}$	$\mathrm {\textsc {crash}}$	
$\mathrm {\textsc {base}}$	74.93	67.09	− 74.33	
$\mathrm {\textsc {ceiling}}$	31.90	39.45	− 24.27	
$\mathrm {\textsc {floor}}$	82.21	63.51	− 55.18	
$\mathrm {\textsc {info}}$	84.75	59.12	− 77.61	

Pairwise MW *U*-tests	$\mathrm {\textsc {rdmax}}$	$\mathrm {\textsc {amplitude}}$	$\mathrm {\textsc {crash}}$	*N*
$\mathrm {\textsc {base}}$ versus $\mathrm {\textsc {ceiling}}$	1.722*	1.280	1.722*	18
$\mathrm {\textsc {base}}$ versus $\mathrm {\textsc {floor}}$	0.866	0.674	− 0.962	17
$\mathrm {\textsc {ceiling}}$ versus $\mathrm {\textsc {floor}}$	− 1.251	− 0.626	0.481	17
$\mathrm {\textsc {base}}$ versus $\mathrm {\textsc {info}}$	0.309	0.221	− 0.221	18

*Top panel:* Treatment medians of peak price ($\mathrm {\textsc {rdmax}}$), price run-ups ($\mathrm {\textsc {amplitude}}$), and price crashes ($\mathrm {\textsc {crash}}$). *Bottom panel:* Significance tests for treatment differences. The numbers indicate *Z*-values of pairwise Mann–Whitney *U*-tests

*, **, and *** represent *p* values smaller than 0.10, 0.05, and 0.01, respectively, for double-sided tests

Given our experimental data, we can also contribute to the growing discussion on the impact of CRT scores and risk aversion on price efficiency and individual trading choices, respectively. In line with Breaban and Noussair (2015), average CRT scores show a negative correlation with overvaluation ($\mathrm {\textsc {rd}}$) at the market level, but the relationship is not significant (Spearman’s $\rho = - 0.21,\, p = 0.227,\, N=35$). Yet, at the individual level, subjects’ CRT scores tend to be negatively related to both price-change beliefs ($\rho = - 0.11,\, p = 0.057,\, N=280$) and asset purchases ($\rho = - 0.20,\, p = 0.001$). Regarding subjects’ average risk aversion in a market, corroborating Crockett et al. (2018), we also observe no significant correlation with overpricing ($\rho$ between 0.06 and 0.17 depending on the measure of risk attitude, all $p > 0.10$, $N=35$). In addition, in contrast to both Breaban and Noussair (2015) and Crockett et al. (2018), we observe no significant correlation between a subject’s risk aversion and either price beliefs or trading behavior (i.e., asset purchases) at the individual level.^9^

Finally, we investigate whether treatment differences are influenced by subject pool variations in risk attitude, gender, CRT score, and other demographics at the treatment level and find no statistically significant differences between treatments.^10^ Thus, we argue that our results are solely driven by the treatment manipulations.

In addition to our analysis of bubble formation, we observe that subjects’ price beliefs translate into trading behavior, i.e., participants who are more optimistic towards future price developments buy significantly more assets prior to the bubble peak than pessimists, which in turn drives market prices (see Appendix B).

## Discussion and conclusion

In recent years, several studies employed experimental asset market environments with a constant $\mathrm {\textsc {fv}}$. Such settings try to replace the decreasing $\mathrm {\textsc {fv}}$ process of the $\mathrm {\textsc {ssw}}$ design, which has been shown to cause confusion about the asset’s $\mathrm {\textsc {fv}}$ and thereby lead to design-induced price inefficiencies. The aim of this paper is to test whether small experimental design manipulations have an impact on the robustness of results in bubble-prone asset markets with a constant $\mathrm {\textsc {fv}}$. In particular, we investigated whether experimental results regarding price efficiency are influenced by (1) changes in the visual representation of the price chart on the trading screen or by (2) providing subjects with complete information about the $\mathrm {\textsc {fv}}$ process. Furthermore, we examine whether beliefs about price developments translate into trading behavior and thus drive prices.

We find that by manipulating the price chart such that the price is displayed at the upper end of the scale, overvaluation can be altered by more than 30 percentage points. Thus, we demonstrate that in a bubble-prone experimental asset market with a constant $\mathrm {\textsc {fv}}$—similar to what Huber and Kirchler (2012), Baghestanian and Walker (2015), and Cason and Samek (2015), among others, have shown for markets with a decreasing $\mathrm {\textsc {fv}}$—seemingly irrelevant design choices can considerably affect market prices. In contrast, we do not find evidence of a change in price efficiency when manipulating the price chart in the opposite direction. To our surprise, overvaluation and bubble formation is not reduced when full information about the $\mathrm {\textsc {fv}}$ process is given. This is at odds with Cheung et al. (2014), who suggest that public knowledge about the $\mathrm {\textsc {fv}}$ encourages price efficiency in an $\mathrm {\textsc {ssw}}$ setting, and is especially puzzling as the reduction of prices due to the seemingly irrelevant manipulation of the price chart would imply confusion among subjects.

Acknowledging the limitations inherent in the explorative nature of this study, however, we want to tread carefully in interpreting our results. We are aware that our experimental design, in which we conduct laboratory asset markets, addresses experimental methodology instead of more general financial markets and entails a comparatively small number of independent observations for testing multiple hypotheses. Thus, we do not claim significance in the sense of type I errors and acknowledge the potential limitations with regard to external validity.

From the results of this study an important implication emerges concerning the design and implementation of constant $\mathrm {\textsc {fv}}$ regimes in financial market experiments: seemingly small variations in the experimental design can actually improve price efficiency, whereas full information about the $\mathrm {\textsc {fv}}$—at least in this setting—does not. These results underpin the fact that further methodological examination is necessary and researchers should be aware of the importance of seemingly small experimental design-features when conducting experimental asset markets.

## Electronic supplementary material

Below is the link to the electronic supplementary material.

Supplementary material 1 (pdf 2272 KB)

## Footnotes

1 See Palan (2013) for a comprehensive survey on markets employing an $\mathrm {\textsc {ssw}}$ design.

2 This design implies an increasing Cash-to-Asset ratio ($\mathrm {\textsc {ca}}$ ratio) over time. However, it is not the aim of this paper to analyze effects of varying $\mathrm {\textsc {ca}}$ ratios over time on market prices. Here, we refer to the broad body of literature examining this within different market environments, e.g., Caginalp et al. (1998), Caginalp et al. (2001), Haruvy and Noussair (2006), Kirchler et al. (2015), Noussair and Tucker (2016), and Razen et al. (2017). Further, Weitzel et al. (2018) investigate the effects of different $\mathrm {\textsc {ca}}$ ratios employing the same basic market design as the present study.

3 Giusti et al. (2016, pp. 45–46) report a more general derivation; Holt et al. (2017) provide a similar intuition relating to rational expectations and risk neutrality.

4 While Haruvy et al. (2007), Kirchler et al. (2015), Razen et al. (2017), among others, elicit price beliefs for all future periods, we follow Holt et al. (2017) with asking subjects to only predict prices in the three following periods.

5 Additional exemplary charts for a maximum price of 28 Taler and 161 Taler, respectively, are provided in Figure A1 in the Appendix.

6 Table A1 in the Appendix outlines details on the calculation. Results are identical when measuring overvaluation by geometric deviation (Powell 2016).

7 This also holds in a regression analysis with binary treatment dummies. The corresponding estimates are provided in Table A2 in the Appendix.

8 Table A1 in the Appendix outlines details on the calculation of each variable.

9 Depending on the specific measure, Spearman’s $\rho$ for correlations between risk-seeking and price-change beliefs (that is, expecting a higher price in the following period(s)) lies between − 0.07 and 0.02. The respective values for correlations between risk-seeking and asset holdings at the end of the experiment lie between − 0.15 and − 0.01; all *p* values $> 0.10$; $N = 280$. The latter, negative (albeit insignificant) correlations imply that a less risk-averse subject will typically hold fewer assets at the end of the experiment.

10 Details on each investigated variable are provided in Table A4 in the Appendix.
