# Supplementary material for: Design-features of bubble-prone experimental asset markets with a constant FV
Source: J Econ Sci Assoc. 2019 Mar 26;5(2):197–209. doi: 10.1007/s40881-019-00061-5 (PMC6917638; doi:10.1007/s40881-019-00061-5)

Appendix to
'Design-features of bubble-prone experimental asset markets with a constant FV'

Christoph Huber, Parampreet Christopher Bindra, Daniel Kleinlercher

A Additional Figures and Tables

Table A1 presents the calculation of all market variables used in the main text.

Table A1: Definitions of market variables.

Measure	Definition
Relative deviation	$\text{RD} = \frac{1}{T}\sum_{t=1}^{T}(\overline{P}_t - FV_t)/\lvert\overline{FV}\rvert$
Share turnover	$\text{ST} = \sum_{t=1}^{T} VOL_t/TSO$
Standard deviation of log-returns	$\text{VOLA} = \sum_{t=1}^{T}\frac{1}{T}\sqrt{\frac{1}{N_t}\sum_{j=1}^{N_t}(R_{t,j} - \overline{R}_t)^2}$
Bid-ask spread at the end of a period	$\text{SPREAD} = \sum_{t=1}^{T}\frac{1}{T}\frac{1}{FV_t}\left[\min_{j\in N_t}\{S_{\hat{t},j}\} - \max_{j\in N_t}\{B_{\hat{t},j}\}\right]$
Maximum relative deviation	$\text{RDMAX} = \text{RD}_{t^*} = \max_t\left\{(\overline{P}_t - FV_t)/FV_t\right\}$
Amplitude	$\text{AMPLITUDE} = \max_t\left\{\frac{\overline{P}_t - FV_t}{FV_t}\right\} - \min_t\left\{\frac{\overline{P}_{t-k} - FV_{t-k}}{FV_{t-k}}\right\}$
Crash	$\text{CRASH} = \min_t\left\{\frac{\overline{P}_t - FV_t}{FV_t}\right\} - \max_t\left\{\frac{\overline{P}_{t+l} - FV_{t+l}}{FV_{t+l}}\right\}$

Notes: $\overline{P}_t$ – (volume-weighted) mean price in period t; FV_t – fundamental value in period t; $\overline{FV}$ – mean fundamental value in the market; T – total number of periods; t^* – peak period (i.e., period with the highest price); $k \in [1, t-1]$ – number of periods before the peak period; $l \in [1, T-1]$ – number of periods after the peak period; log-return of a trade: $R_{t,j} = ln(P_{t,j}/P_{t,j-1})$; total number of trades in period t: N_t; average log return in period t: $\overline{R}_t$; price of sell order j at the end of period t: $S_{\hat{t},j}$; price of buy order j at the end of period t: $B_{\hat{t},j}$. All variables above are calculated on the market level.

Figure A1 shows exemplary price charts for maximum prices of 28 (top), 51 (middle), or 161 (bottom) for treatments BASE and INFO (left), CEILING (middle), and FLOOR (right).

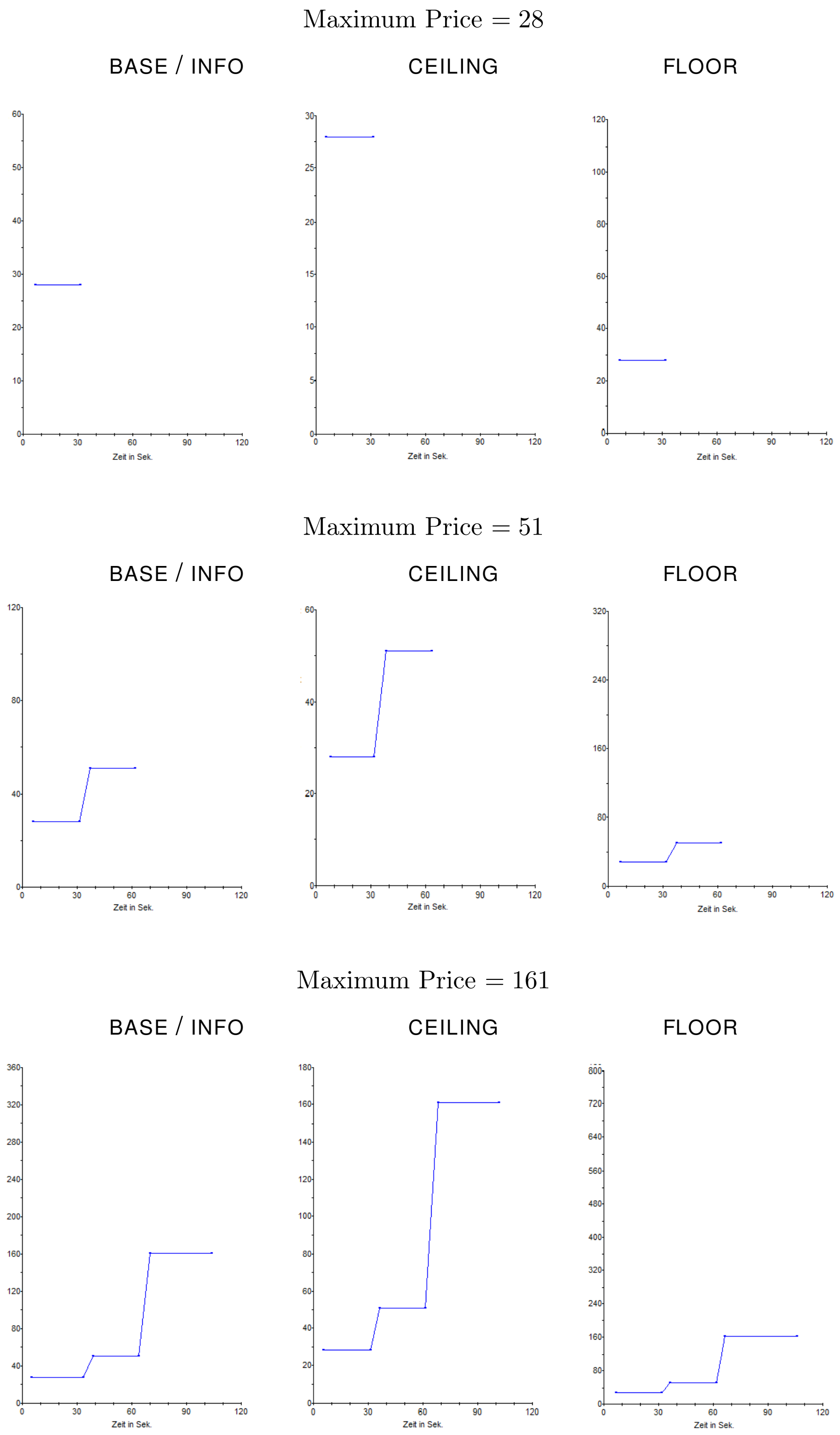

Figure A1: Exemplary price charts on the trading screen.

Figure A2 presents the evolution of mean treatment prices (solid gray lines) and the mean price forecasts (black lines with triangles) for periods $t + k$ with $k = 0, 1, 2$ for each of the four treatments.

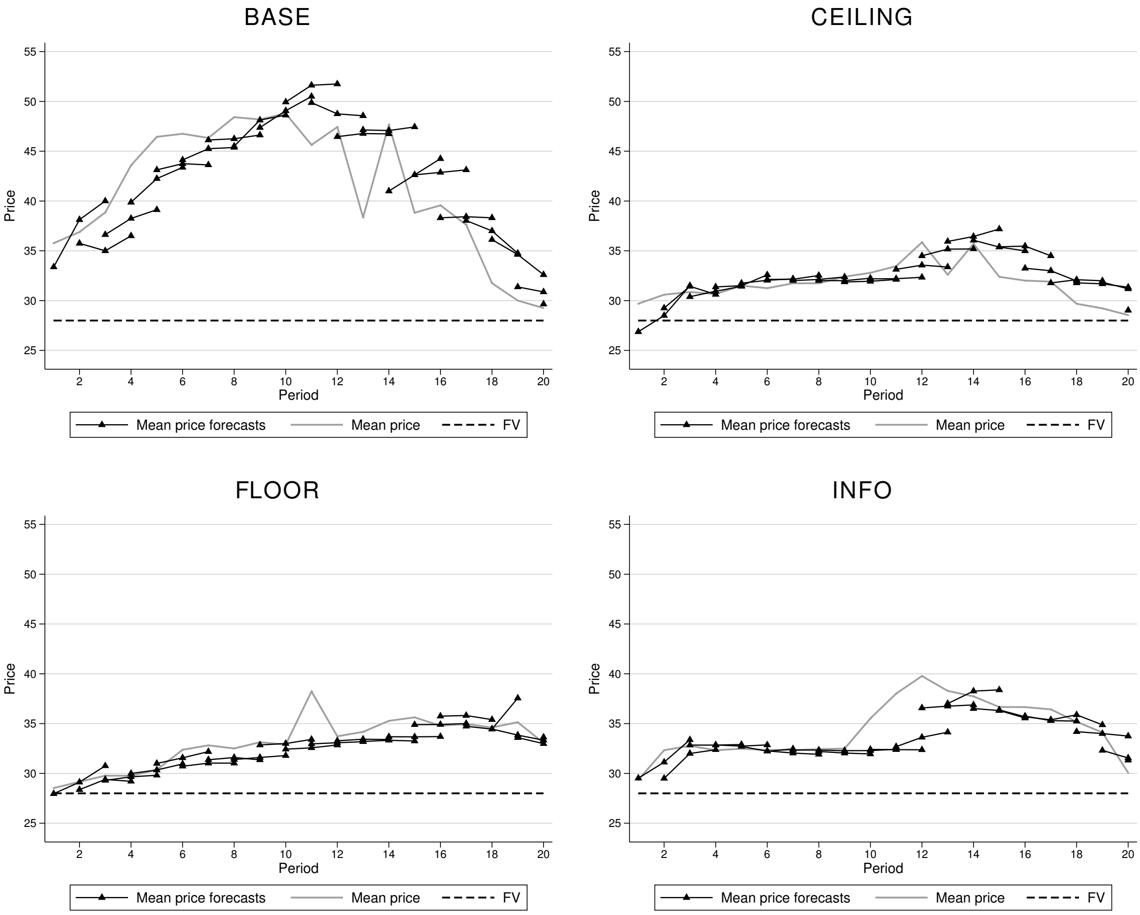

Figure A2: Median beliefs about market prices (black lines with triangles) up to period $t + 2$, as a function of period for BASE (top left), CEILING (top right), FLOOR (bottom left), and INFO (bottom right). The median treatment price is depicted as grey solid line. The dashed line shows the fundamental value (FV) of 28. One observation with implausible price beliefs of 3,000 Taler was dropped in Treatment FLOOR.

Table A2: Random-effects regressions for differences in market variables across treatments with clustered standard errors on the market level.

	RD	ST	VOLA	SPREAD
α	0.772***	0.174***	0.082***	0.210***
	(3.462)	(5.391)	(8.208)	(5.345)
CEILING	−0.474**	0.086	0.003	0.009
	(−1.963)	(1.047)	(0.112)	(0.086)
FLOOR	−0.244	0.038	0.028	0.184
	(−0.772)	(0.768)	(1.101)	(1.209)
INFO	0.176	0.036	0.064	0.249
	(0.327)	(0.679)	(1.194)	(1.025)
N	699	700	696	674

Pairwise Wald-tests:

CEILING VS. FLOOR	0.90	0.32	0.48	1.03
CEILING VS. INFO	1.70	0.33	1.06	0.88
FLOOR VS. INFO	0.61	0.00	0.40	0.05

Notes: Dependent variables: Overvaluation (RD) is the percentage deviation of prices from the fundamental value (FV). Share turnover (ST) is applied as a measure of trading volume. The standard deviation of log-returns (VOLA) serves as a proxy for volatility. The bid-ask-spread at the end of a period is calculated as a percentage of the FV in the market (SPREAD). z-statistics are given in parentheses. CEILING, FLOOR, and INFO are binary treatment dummies showing 1 for the respective treatment and 0 otherwise. Top panel: coefficient values with corresponding z-statistics (in parentheses) are provided. Bottom panel: χ^2-statistics of pairwise Wald-tests are shown. Non-parametric, pairwise Mann-Whitney U-tests on the market level yield qualitatively very similar results (see Table 1 in the Appendix).

In Table A3 we provide details on individual market results for each bubble measure in all four treatments.

Table A3: Individual market results for bubble measures overvaluation (RD; ?) as well as RDMAX, AMPLITUDE, and CRASH (?).

Treatment	Market	RD	RDMAX	AMPLITUDE	CRASH
BASE	1	229.34	632.31	601.84	−625.46
	2	58.24	159.52	151.21	−155.95
	3	44.93	72.90	44.29	−69.59
	4	161.75	288.57	164.46	−284.03
	5	32.87	68.48	67.09	−31.99
	6	43.50	73.34	40.90	−63.82
	7	47.32	74.93	66.62	−74.33
	8	28.43	59.37	38.93	−55.43
	9	48.20	105.61	87.36	−101.17
	Mean	77.17	170.56	140.30	−162.42
	Median	47.32	74.93	67.09	−74.33
CEILING	1	13.46	15.74	6.89	−3.21
	2	79.74	196.07	204.62	−207.30
	3	28.91	63.32	39.45	−65.31
	4	33.33	90.69	91.72	−65.12
	5	3.30	6.04	0.00	−5.75
	6	16.91	31.90	61.56	−24.27
	7	12.78	15.68	1.68	−13.76
	8	75.91	325.69	310.94	−329.87
	9	3.77	7.29	10.89	−6.19
	Mean	29.79	83.60	80.86	−80.09
	Median	16.91	31.90	39.45	−24.27
FLOOR	1	15.35	35.33	0.00	−31.24
	2	89.90	174.87	166.04	−150.39
	3	11.93	17.36	16.86	−1.55
	4	6.85	19.21	30.79	−3.48
	5	202.60	1009.13	1010.59	−1015.82
	6	19.61	99.52	122.77	−122.77
	7	22.90	98.64	96.23	−79.13
	8	53.25	65.79	26.72	−26.72
	Mean	52.80	189.98	183.75	−178.89
	Median	21.26	82.21	63.51	−55.18
INFO	1	16.36	59.88	59.12	−59.12
	2	35.65	118.19	118.91	−114.71
	3	64.27	199.84	88.23	−208.34
	4	14.88	17.96	2.82	−19.09
	5	25.24	38.98	42.13	−23.68
	6	144.24	910.12	907.85	−907.01
	7	14.32	31.57	31.14	−22.33
	8	488.76	1007.14	967.47	−778.57
	9	49.14	84.75	47.25	−77.61
	Mean	94.76	274.27	251.66	−245.61
	Median	35.65	84.75	59.12	−77.61

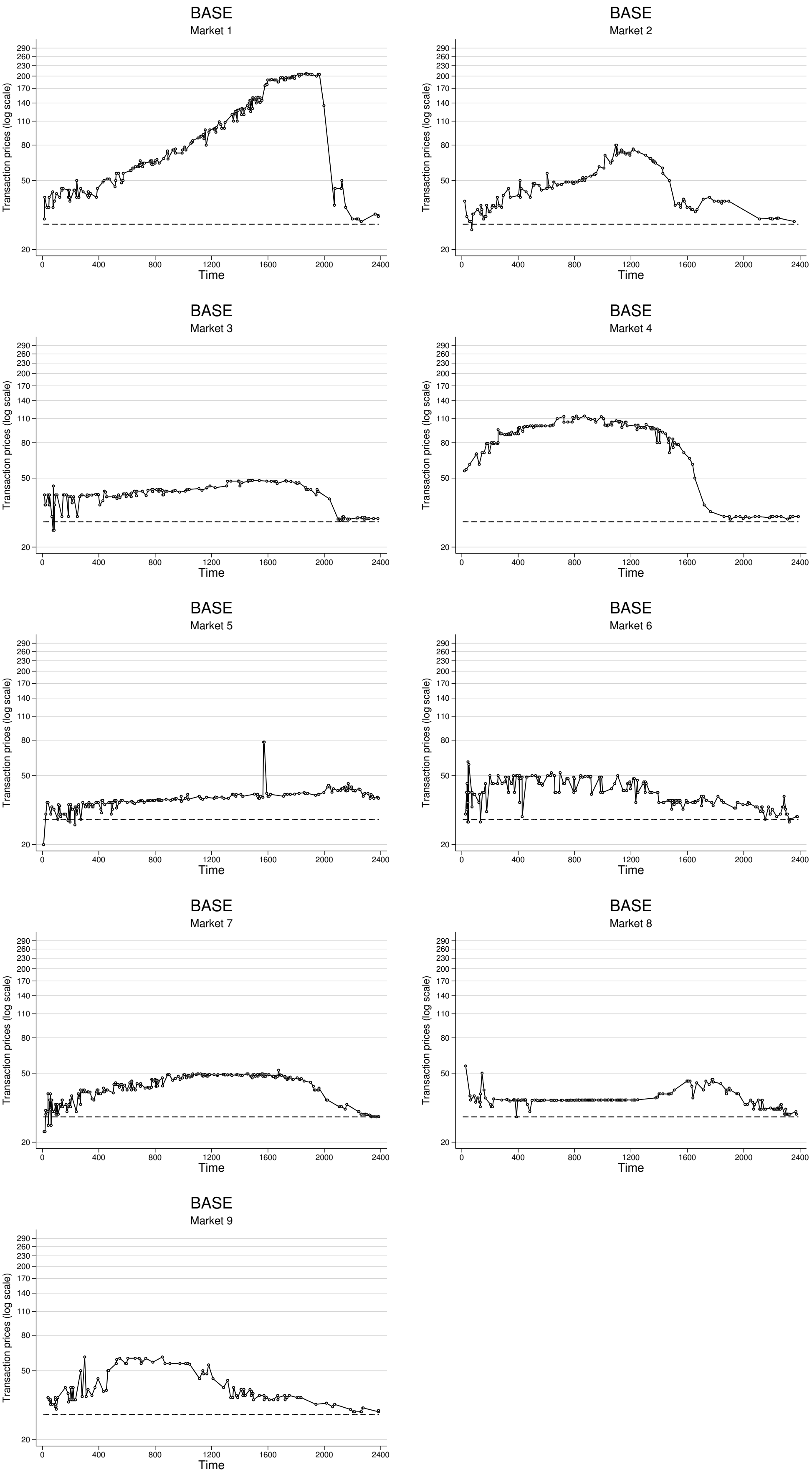

Figure A3: Individual transaction prices for each market of Treatment BASE. The dashed line represents the risk-neutral fundamental value of 28.

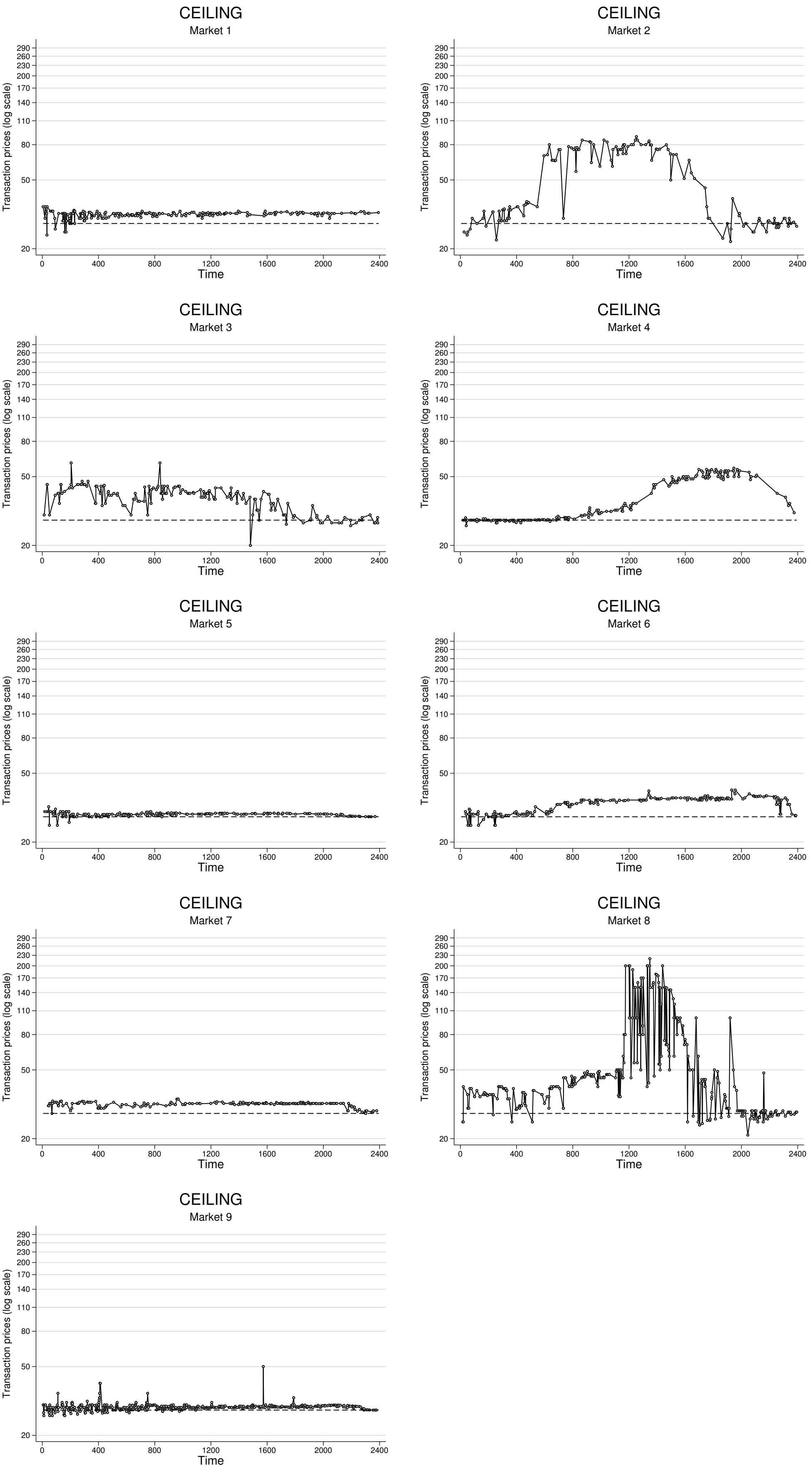

Figure A4: Individual transaction prices for each market of Treatment CEILING. The dashed line represents the risk-neutral fundamental value of 28.

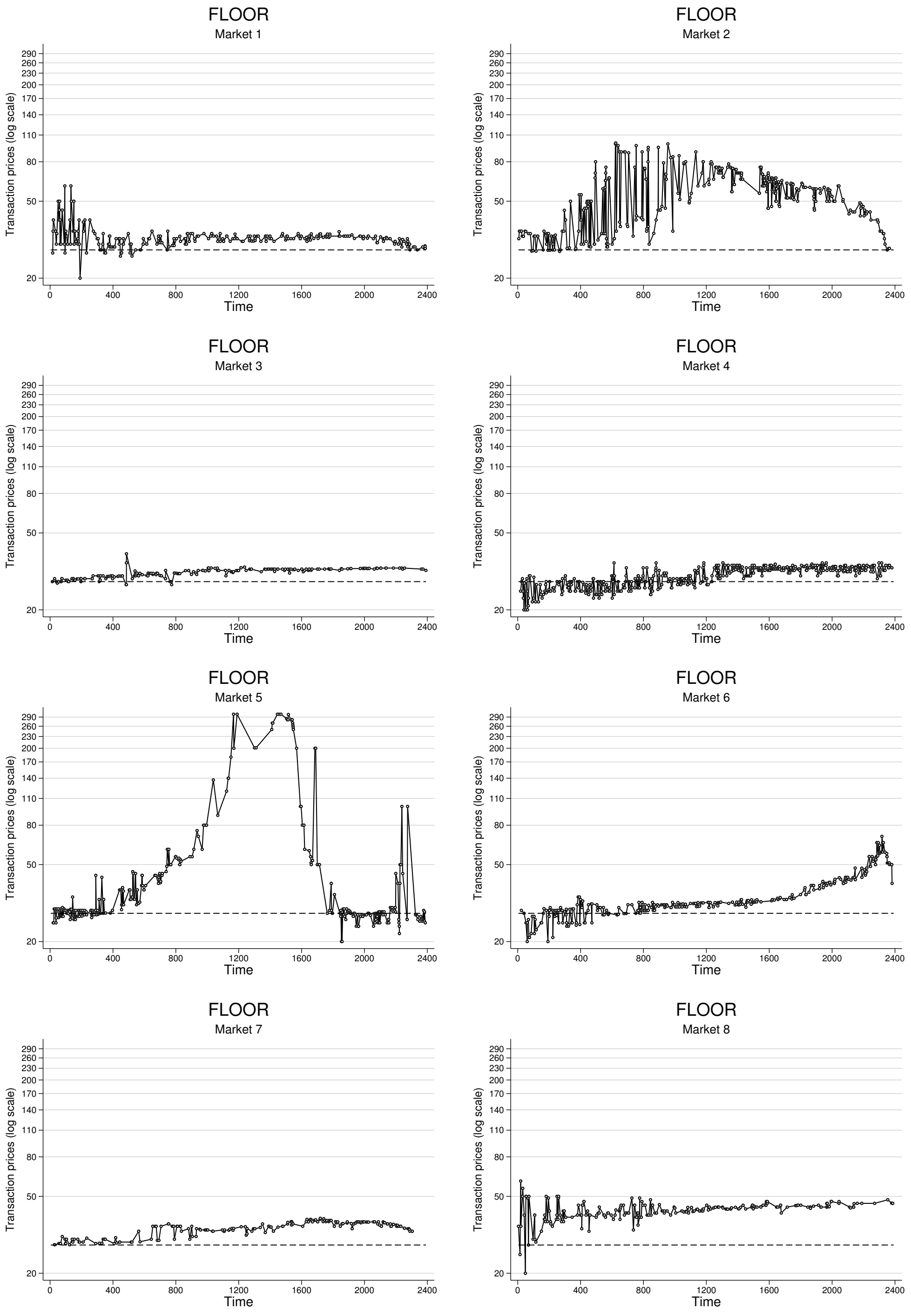

Figure A5: Individual transaction prices for each market of Treatment FLOOR. The dashed line represents the risk-neutral fundamental value of 28.

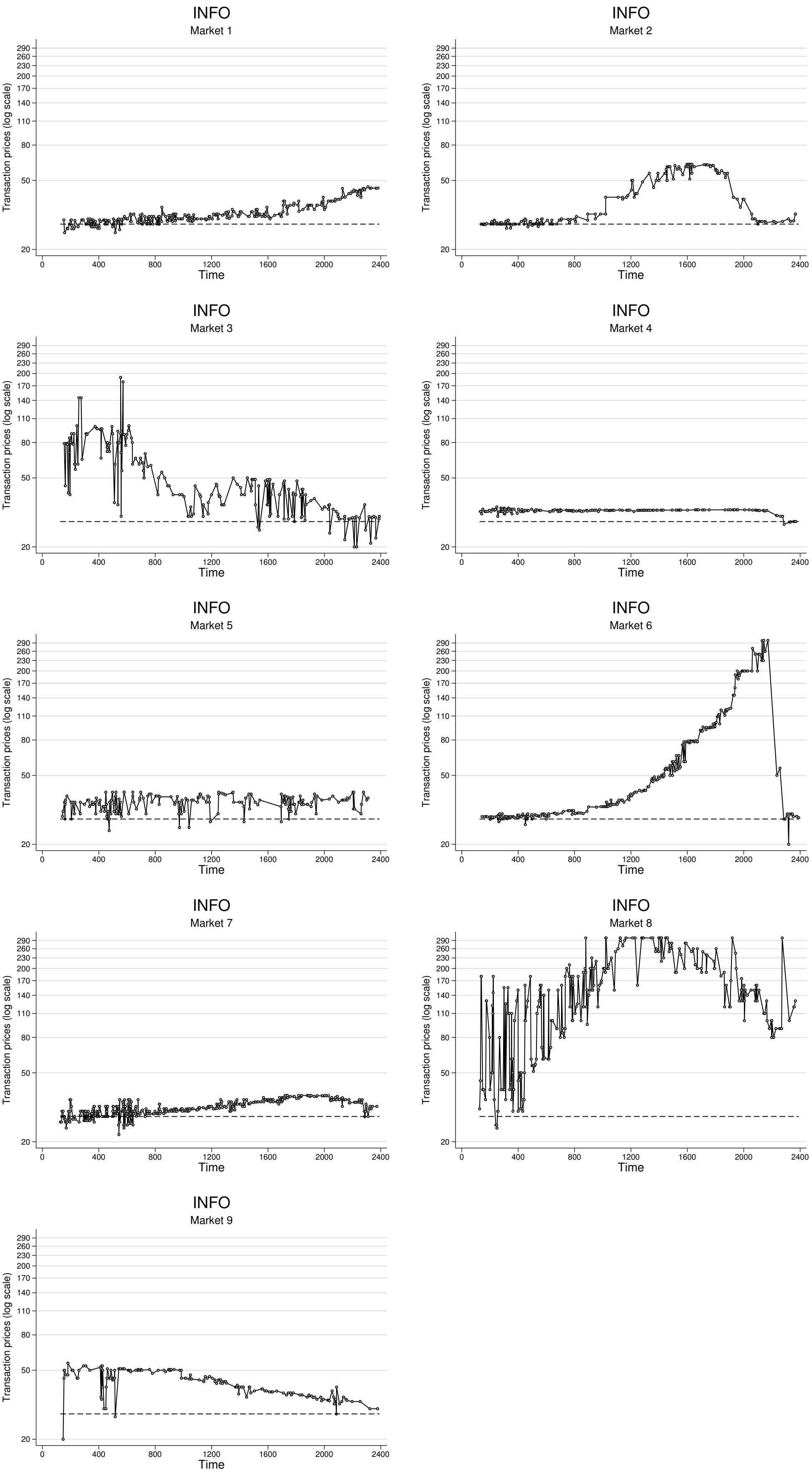

Figure A6: Individual transaction prices for each market of Treatment INFO. The dashed line represents the risk-neutral fundamental value of 28.

In Table A4 summarizes subject demographics between all four treatments and p-values for differences across treatments.

Table A4: Subject demographics by treatment.

	BASE		CEILING		FLOOR		INFO		p-value
Female (%)	40.28	(49.39)	40.28	(49.39)	51.56	(50.37)	36.11	(48.37)	0.31
Age	23.58	(2.98)	22.79	(2.68)	23.75	(3.77)	23.21	(3.38)	0.38
Semesters of Study	6.22	(3.45)	5.43	(3.55)	6.44	(4.82)	5.63	(3.99)	0.42
Experience	1.94	(1.27)	1.76	(1.40)	2.20	(1.34)	1.57	(1.41)	0.05
Risk attitude general	5.00	(2.30)	4.96	(2.32)	5.34	(2.46)	5.13	(2.31)	0.77
Risk attitude investments	4.06	(2.19)	4.04	(2.46)	4.27	(2.45)	4.06	(2.23)	0.91
CRT score	1.88	(1.15)	1.64	(1.10)	1.77	(1.32)	1.68	(1.24)	0.62
N	72		72		72		64		

Notes: This table represents mean values of different subject demographics across treatments. Standard deviations are shown in parentheses. *Female* represents the percentage share of female subjects; *Age* is subjects' age in years; *Semesters of study* is the average of subjects' study time in semesters; *Experience* represents subjects' experience with participating in laboratory experiments (0 = "This is my first experiment.", 1 = "1 to 5 experiments", 2 = "6 to 15 experiments", 3 = "more than 16 experiments"); *Risk attitude general* is the average value to the question "In general, are you willing to take of to you avoid taking risky decisions?" [Likert-scale ranging from 0 (avoid taking risky decisions) to 10 (willing to take risky decisions)]; *Risk attitude investments* is the average value to the question "With respect to investments, are you willing to take of to you avoid taking risky decisions?" [Likert-scale ranging from 0 (avoid taking risky decisions) to 10 (willing to take risky decisions)]; *CRT score* is the number of correct answers (out of 4) of a 4-question Cognitive Reflection Test (**?**) as proposed by **?**. The column "p-values" reprents p-values from Kruskal-Wallis equality-of-populations rank tests between treatments.

B Additional Results: Beliefs about Prices and Trading Behavior

In each period, we elicited subjects' beliefs about the average market price in the following three periods. This procedure allows us to analyze how participants' expectations about future price developments relate to trading behavior and thus drive market prices.

Result B1. We detect strong beliefs about increasing prices across all subjects in treatments BASE and FLOOR, as well as for the most optimistic traders across all treatments.

Support: Following **?** and **?**, we run least squares regressions with market-fixed effects to detect belief dynamics. We calculate differences between subjects' price beliefs and past period prices ($\overline{P}_{m,t-1}$) according to the following equations,

$$\overline{BeP}_{m,t,t+k} = \overline{F}_{m,t,t+k} - \overline{P}_{m,t-1}; \tag{4}$$

$$BeP^{\text{OPT}}_{m,t,t+k} = \text{OPT}(F_{m,t,t+k}) - \overline{P}_{m,t-1}; \qquad \text{with } k = 0, 1, 2; \tag{5}$$

where $\overline{F}_{m,t,t+k}$ is the average belief for period $t+k$, elicited in period t, among all subjects in market m (BeP is an acronym for "Beliefs about Prices"). $\text{OPT}(F_{m,t,t+k})$ describes the optimists' beliefs – that is, the second-highest price belief (85-percentile) in a market. We then subtract the past period's average market price $\overline{P}_{m,t-1}$ and estimate the following regression model:

$$y_{m,t,t+k} = \alpha + \epsilon_{m,t}; \qquad k = 0, 1, 2 \tag{6}$$

with $y_{m,t,t+k}$ being a generic placeholder for either $\overline{BeP}_{m,t,t+k}$ or $BeP^{\text{OPT}}_{m,t,t+k}$. As we aim to explain differences between treatments in the price run-ups, we restrict our analysis in this part to periods before or at the peak period in each market, i.e., $t \leq t^*$, and test each treatment separately for significance. A positive intercept indicates beliefs about future prices being higher than last period's average market price, i.e., subjects expect prices to further increase in the next periods.

Table A5: Market-fixed effects panel regression of Eq. (6): Beliefs about Prices.

	$\overline{BeP}_{m,t,t+k}$			$BeP^{\text{OPT}}_{m,t,t+k}$				
	t	$t+1$	$t+2$	t	$t+1$	$t+2$	N	FE
BASE	1.784***	3.056***	4.150***	4.725***	7.090***	9.628***	92	CS
	(0.256)	(0.318)	(0.407)	(0.383)	(0.497)	(0.737)		
CEILING	0.021	0.622	1.002**	3.081***	3.602***	4.842***	75	CS
	(0.524)	(0.420)	(0.444)	(0.825)	(0.675)	(0.701)		
FLOOR	0.578	1.374***	2.134***	4.191***	6.395***	8.820***	101	CS
	(0.460)	(0.506)	(0.734)	(1.014)	(1.505)	(2.379)		
INFO	−0.173	0.620	0.828	5.446***	6.998***	8.891***	92	CS
	(0.731)	(0.770)	(0.695)	(1.417)	(1.821)	(2.213)		

Notes: $\overline{BeP}_{m,t,t+k}$ detects speculative motives by comparing average beliefs about future market prices up to $t+2$, elicited in period t (market average), with the average market price of the last period. $BeP^{\text{OPT}}_{m,t,t+k}$ applies only the two highest beliefs in each period. In each market, only periods before or at the price peak, $t \leq t^*$ are considered; this leads to different sample sizes across treatments. *, **, and *** represent double-sided tests' p-values smaller than .10, .05, and .01. One observation with implausible price beliefs of 3,000 Taler was dropped in Treatment FLOOR.

In the left panel of Table A5, showing the estimation results with average beliefs ($\overline{BeP}_{m,t,t+k}$), we observe positive intercepts in treatments BASE and FLOOR which indicates that subjects expect prices to increase. However, **?** and **?** show that the most optimistic traders' beliefs in each period ($BeP^{\text{OPT}}_{m,t,t+k}$) are the ones who drive market prices. Estimating the same model in Eq. (6) for optimists' beliefs only, we find that beliefs are considerably higher than last period's prices in all treatments (see right panel of Table A5). Testing for differences between treatments in multivariate regressions with treatment dummies, we find that belief dynamics regarding optimists are statistically indistinguishable between treatments. Considering all subjects, we only observe marginally lower values of $\overline{BeP}_{m,t,t+k}$ in INFO than in BASE.[11]

As a rationality check, to test whether the price beliefs of the most optimistic/pessimistic traders also translate into trading behavior, we investigate the change of asset holdings per period between optimists and pessimists prior to the peak period of each market. Across all treatments, we find that optimists buy more assets prior to the bubble peak than pessimists. Thus, optimists' price beliefs do translate into trading behavior and, therefore, drive prices.[12]

[11]The estimated coefficients and pairwise tests between treatments are provided in Table A6 in the Appendix.

[12]See Table A7 and the related discussion in the Appendix for a more detailed analysis.

Table A6 shows estimates from random effects regressions on Beliefs about Prices with treatment dummies (top panel) as well as pairwise Wald-tests for differences between treatments (bottom panel).

Table A6: Random effects panel regression for differences in Beliefs about Prices between treatments.

	$\overline{BeP}_{m,t,t+k}$			$BeP^{\text{OPT}}_{m,t,t+k}$		
	t	$t+1$	$t+2$	t	$t+1$	$t+2$
α	1.785*	3.131***	4.328***	5.008***	7.426***	10.19***
	(0.928)	(1.141)	(1.473)	(1.716)	(2.416)	(3.304)
CEILING	−1.814	−2.465	−3.230	−1.758	−3.451	−4.841
	(1.402)	(1.717)	(2.208)	(2.596)	(3.648)	(4.990)
FLOOR	−1.093	−1.432	−1.744	0.0418	0.336	0.205
	(1.364)	(1.691)	(2.196)	(2.514)	(3.557)	(4.874)
INFO	−2.540*	−3.114*	−4.297**	0.915	−0.166	−1.080
	(1.346)	(1.653)	(2.128)	(2.490)	(3.505)	(4.797)
N	360	357	353	360	357	353
Pairwise Wald-tests:						
CEILING vs. FLOOR	0.25	0.33	0.41	0.45	1.00	0.95
CEILING vs. INFO	0.26	0.14	0.22	1.01	0.77	0.54
FLOOR vs. INFO	1.07	0.95	1.30	0.11	0.02	0.07

Notes: $\overline{BeP}_{m,t,t+k}$ detects speculative motives by comparing average beliefs about future market prices up to $t+2$, elicited in period t (market average), with the average market price of the last period. $BeP^{\text{OPT}}_{m,t,t+k}$ applies only the two highest beliefs in each period. In each market, only periods before or at the price peak, $t \leq t^*$ are considered. CEILING, FLOOR, and INFO are binary treatment dummies showing 1 for the respective treatment and 0 otherwise. Top panel: coefficient values with corresponding z-statistics (in parentheses) are provided. Bottom panel: χ^2-statistics of pairwise Wald-tests are shown. One observation with implausible price beliefs of 3,000 Taler was dropped in Treatment FLOOR.

As a rationality check, we run ordinary least squares regressions for differences in period-to-period changes in asset holdings between optimists and pessimists in all pre-peak periods separately for each treatment (see Table A7). In particular, we regress the change in asset holdings per period on the binary variable OPTIMIST indicating 1 for optimists and 0 for pessimists.[13] We find significantly positive coefficients in each treatment with the highest value for BASE. Here, we estimate that optimists buy on average 0.80 assets per period while pessimists sell on average 1.09 assets. Thus, we find that having comparatively optimistic price beliefs is conducive to buying more assets than subjects with comparatively pessimistic beliefs. In price run-ups, optimists are indeed the ones who drive prices upwards.

Table A7: Ordinary least squares regression for differences in period-to-period changes in asset holdings between optimists and pessimists up to the price peak in each market.

	BASE	CEILING	INFO	FLOOR
OPTIMIST	1.896***	1.495**	1.289**	0.772**
	(0.541)	(0.678)	(0.576)	(0.377)
α	−1.094***	−0.391	−0.651***	−0.738***
	(0.184)	(0.383)	(0.236)	(0.260)
N	404	341	436	407

Notes: Dependent variable: Period-to-period change in asset holdings. OPTIMIST is a dummy variable indicating whether a subject is among the two most optimistic traders – that is, whether her price forecasts are on average among the two *highest* – in her respective market across all pre-peak periods. If OPTIMIST $= 0$ the respective subject's price forecasts are on average among the two *lowest* across all pre-preak periods and she is therefore classified as a pessimist. Clustered standard errors on the subject-level are provided in parentheses. *, **, and *** represent double-sided tests' p-values smaller than .10, .05, and .01.

[13] This variable is not defined for the remaining four subjects in a market who are neither classified as optimists nor as pessimists.

C Instructions of the Experiments

All experimental studies and supplementary questionnaires described in this paper were conducted in German. In the following we display the translated versions. The original versions in German language are available upon request.

C.1 Experimental Instructions of the Bomb Risk Elicitation Task

Description of the Game

This experiment is designed to evaluate economic decision making. Your task is to indicate which of the 25 boxes on the decision-screen (on the next page), which are arranged in five rows and five columns, you would like to select. Under one of these boxes will be placed a "bomb" by random mechanism at the beginning of the experiment. The box under which the bomb is, is determined for each participant individually. The other boxes are empty. You do not know under which box the bomb is. You only know that the bomb could be hidden equally likely under each box.

By clicking a box you can mark it (it changes color and turns white) to open it. You can mark as many boxes as you like. Would you change your decision, you can deselect a marked box with a simple click on it. This procedure can be repeated as often as you like. After you have marked the desired boxes, confirm your selection with the "Confirm" button. You have 240 seconds to decide.

Payment

Your payment is dependent on whether the bomb is under a selected box or not. If the bomb is not under a box which you selected, you get a payment of 40 Cents per selected box, but if the bomb is under a box you will not get a payment for this experiment.

Information for your payment

The reversing of the boxes will be after the second experiment. You will see the screen with your selected boxes again and with a click on "reverse selected boxes" you will see if you have selected the box with the bomb (indicated by red color). You will see your total payment of this experiment on the bottom right of the screen.

Important Information

- You can select and deselect boxes as often as you like.

- You have 240 seconds for your decision.
- The reversing of the boxes will be after the second experiment.

Screenshot

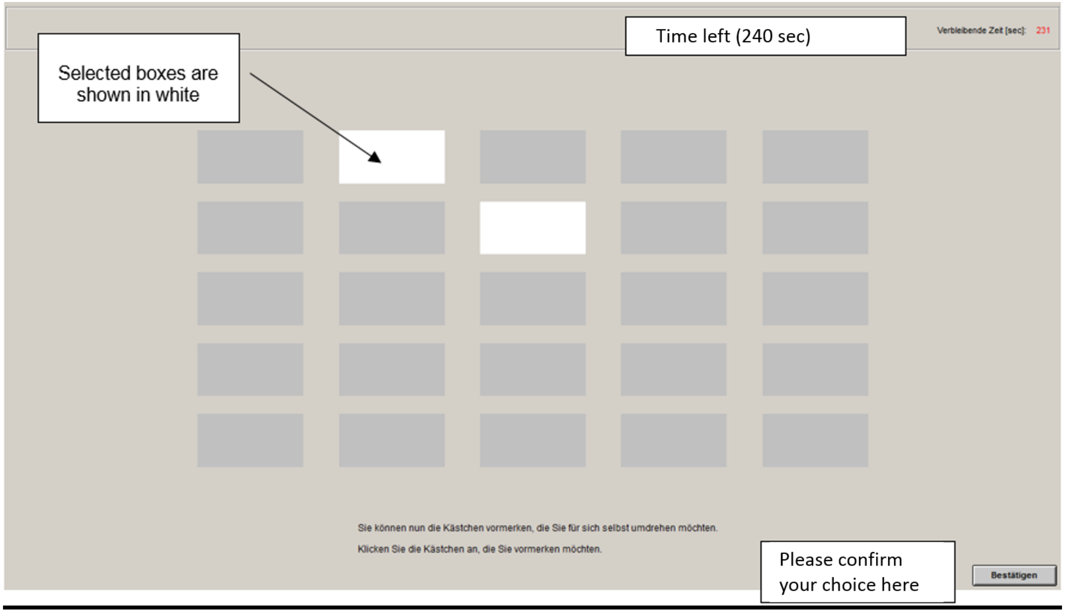

C.2 Experimental Instructions for the Market Experiment

In the following, text parts included only in Treatment INFO are in *italics*. Text parts in standard font are identical for all treatments.

Background of the experiment

This experiment replicates an asset market in which 8 traders can trade assets of a fictitious company over 20 periods, where each period lasts for 120 seconds. You receive an initial endowment of 20 assets and 560 Taler (experimental currency, converted to EUR at the end of the experiment). Your asset and Taler holdings carry over from one period to the next. Your asset and Taler holdings cannot drop below zero.

To familiarize you with the software and the trading mechanism, there will be 2 trial periods, which are not relevant for your final payment.

Information on the market architecture and your tasks as a trader

1) Trading

Participating in the market as a trader you can sell and buy assets. Trade is accomplished in form of a continuous double auction. That is, every trader can buy as well as sell assets. You can submit as many buy and sell orders (with at most 2 decimal places) as you like. You have to specify the number of stocks you want to trade for every order.

If you buy assets, your Taler holdings will be decreased by the respective expenditures (price x quantity) and the number of assets will be increased by the quantity of newly bought assets. Inversely, if you sell assets, your Taler holdings will be increased by the respective revenues (price x quantity) and the number of assets will be decreased by the quantity of newly sold assets. Please note that you can only buy (sell) as many assets as are covered by your Taler (asset) holdings – this includes also your active offers in the market.

Each share held at the of a trading period will pay a dividend, which amounts to 1.20 Taler or 1.60 Taler per share with equal probability. The randomly selected dividend is the same for each share and is newly determined each period. Additionally, you receive interest payments of 5% on your current Taler holdings. These dividend and interest payments are added to your Taler holdings.

At the end of the experiment the units you own are bought back by the experimenter at a buyback price of 28 Taler per share.

At the beginning of each new period you receive an income of 100 Taler. This will be added to your Taler holdings.

The following table might help you making decisions. It shows the development of the fundamental value of an asset. This is calculated from the interest rate, dividends, and the buyback price of 28. In column "Current period" you see the period which corresponds to the calculation of the fundamental value in this line. Column "Average dividend value per period" shows the average amount, which can be expected from dividend payments per share and per period. The average dividend value remaining is calculated by multiplying the average dividend value per period and the number of remaining periods. Column "Average interest payments up to this period" shows the average interest payments, which you would have accumulated, if you bought a share not until this period. Hence, the last column presents the thereby calculated fundamental value of a share. It follows from multiplying "Number of periods remaining" and "Average dividend value per period" and then adding the "Average interest payments up to this period".

Current period	*Number of holding periods*	*Average dividend value per period*	*Average dividends remaining*	*Average interest payments up to this period*	*Fundamental value per unit of inventory*
1	*20*	*1.40*	*28.00*	*0.00*	*28.00*
2	*19*	*1.40*	*26.60*	*1.40*	*28.00*
3	*18*	*1.40*	*25.20*	*2.80*	*28.00*
4	*17*	*1.40*	*23.80*	*4.20*	*28.00*
5	*16*	*1.40*	*22.40*	*5.60*	*28.00*
6	*15*	*1.40*	*21.00*	*7.00*	*28.00*
7	*14*	*1.40*	*19.60*	*8.40*	*28.00*
8	*13*	*1.40*	*18.20*	*9.80*	*28.00*
9	*12*	*1.40*	*16.80*	*11.20*	*28.00*
10	*11*	*1.40*	*15.40*	*12.60*	*28.00*
11	*10*	*1.40*	*14.00*	*14.00*	*28.00*
12	*9*	*1.40*	*12.60*	*15.40*	*28.00*
13	*8*	*1.40*	*11.20*	*16.80*	*28.00*
14	*7*	*1.40*	*9.80*	*18.20*	*28.00*
15	*6*	*1.40*	*8.40*	*19.60*	*28.00*
16	*5*	*1.40*	*7.00*	*21.00*	*28.00*
17	*4*	*1.40*	*5.60*	*22.40*	*28.00*
18	*3*	*1.40*	*4.20*	*23.80*	*28.00*
19	*2*	*1.40*	*2.80*	*25.20*	*28.00*
20	*1*	*1.40*	*1.40*	*26.60*	*28.00*

Because of the value of dividend payments and discounting the fundamental value is 28 Taler in each period. Note that the fundamental value is constant over all periods.

Example for the calculation of the fundamental value: Suppose there are four trading periods remaining. As the dividend per share is 1.20 Taler and 1.60 Taler, respectively, with 50% probability each, the expected dividend payment is 1.40 Taler per share and per period. Suppose you own just one share and hold it until the end of the market. Then you can expect dividend payments of $4 \times 1.40 = 5.60$ *Taler for the remaining four periods. If you buy this share not until this period, you have accumulated interest payments for the value of the share in the previous 16 periods amounting to* $28 \times 0.05 = 1.40$ *each, hence* $16 \times 1.40 = 22.40$*. The fundamental value of a share in this period is therefore the sum:* $5.60 + 22.40 = 28$ *Taler.*

Example for the calculation of the dividend and your asset and Taler holdings: Suppose you begin the experiment with 560 Taler in cash and 20 assets. If you make no purchases or sales, the interest earnings will be 28 Taler, that is $560 \times 0.05 = 28$ Taler. If the randomly determined dividend turns out to be 1.20 Taler, the total dividend income will be $20 \times 1.20 = 24$ Taler. These $28 + 24 = 52$ Taler, as well as your income of 100 Taler, will be added to your Taler holdings at the end of the period. Hence, your initial endowment at the beginning of

the next period will be 6 assets and 712 Taler ($560 + 52 + 100$).

2) Predictions

Additionally to your trading activity you will be asked to predict the development of market prices over the three subsequent periods. Exceptions are the penultimate period with two predictions and the last period with one prediction.
If your prediction is within +- 5% of the average market price in the corresponding period, your earnings from the three predictions are 50 Taler each. That is, you can earn a maximum of 150 Taler for your predictions in each period. These earnings will be added to your Taler holdings at the end of the last period.

Calculation of your payment

At the end of the experiment, your payment as a trader is calculated as follows:
The number of assets you hold are bought back by the experimenter at the end of the experiment (after period 20). You will receive 28 Taler for each asset you hold. The total amount is added to your final cash (Taler) holdings. Additionally, your earnings from all your predictions will be added to your Taler holdings.

Final Wealth in Taler = asset holdings * 28 Taler + Taler holdings
+ income from market predictions

Your earnings from this experiment will then be converted to EUR using a conversion rate of 1 Euro for 400 Taler.

Final Wealth in EUR = Final Wealth in Taler / 400 Taler

Trading Screen

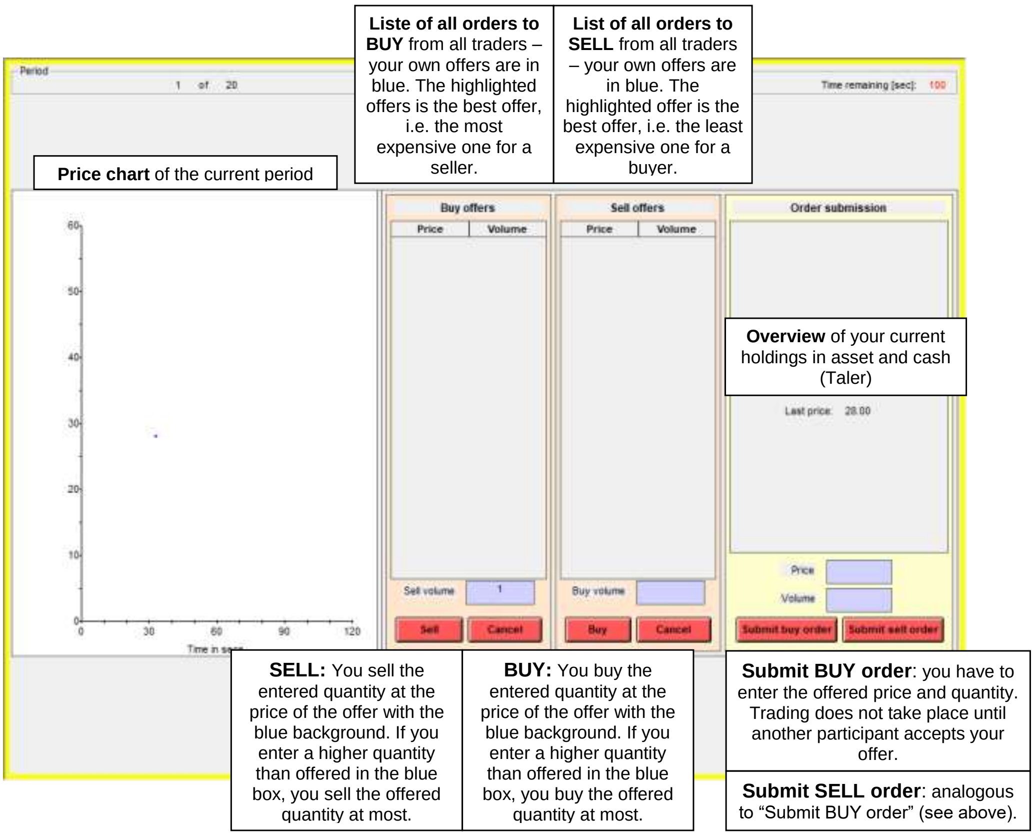

History Screen

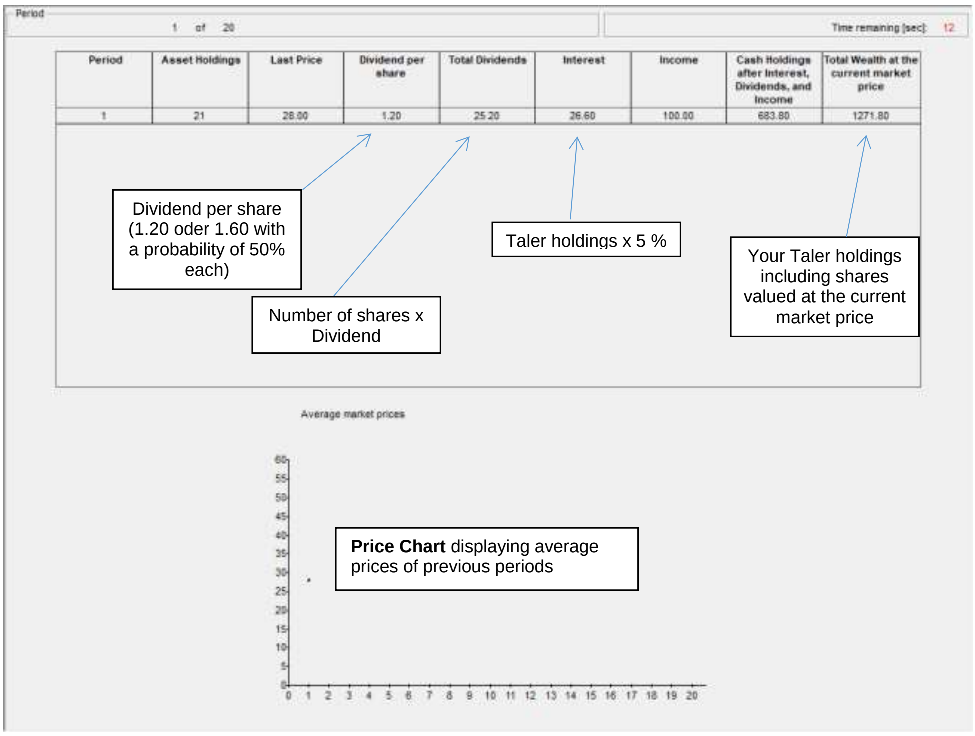

Supplement: Supplementary file 1 — Supplementary material 1 (pdf 2272 KB) [file 40881_2019_61_MOESM1_ESM.pdf]
